# Microsomal and Cytosolic Scaling Factors in Dog and Human Kidney Cortex and Application for In Vitro-In Vivo Extrapolation of Renal Metabolic Clearance[Fn FN4]

**DOI:** 10.1124/dmd.117.075242

**Published:** 2017-05

**Authors:** Daniel Scotcher, Sarah Billington, Jay Brown, Christopher R. Jones, Colin D. A. Brown, Amin Rostami-Hodjegan, Aleksandra Galetin

**Affiliations:** Centre for Applied Pharmacokinetic Research, University of Manchester, Manchester (D.S., A.R.-H., A.G.); Newcastle University, Newcastle (S.B., C.D.A.B.); Biobank, Central Manchester University Hospitals NHS Foundation Trust, Manchester (J.B.); DMPK, Oncology iMed, AstraZeneca R&D, Alderley Park, Macclesfield (C.R.J.); and Simcyp Limited (a Certara Company), Blades Enterprise Centre, Sheffield (A.R.-H.), United Kingdom

## Abstract

In vitro-in vivo extrapolation of drug metabolism data obtained in enriched preparations of subcellular fractions rely on robust estimates of physiologically relevant scaling factors for the prediction of clearance in vivo. The purpose of the current study was to measure the microsomal and cytosolic protein per gram of kidney (MPPGK and CPPGK) in dog and human kidney cortex using appropriate protein recovery marker and evaluate functional activity of human cortex microsomes. Cytochrome P450 (CYP) content and glucose-6-phosphatase (G6Pase) activity were used as microsomal protein markers, whereas glutathione-S-transferase activity was a cytosolic marker. Functional activity of human microsomal samples was assessed by measuring mycophenolic acid glucuronidation. MPPGK was 33.9 and 44.0 mg/g in dog kidney cortex, and 41.1 and 63.6 mg/g in dog liver (*n* = 17), using P450 content and G6Pase activity, respectively. No trends were noted between kidney, liver, and intestinal scalars from the same animals. Species differences were evident, as human MPPGK and CPPGK were 26.2 and 53.3 mg/g in kidney cortex (*n* = 38), respectively. MPPGK was 2-fold greater than the commonly used in vitro-in vivo extrapolation scalar; this difference was attributed mainly to tissue source (mixed kidney regions versus cortex). Robust human MPPGK and CPPGK scalars were measured for the first time. The work emphasized the importance of regional differences (cortex versus whole kidney–specific MPPGK, tissue weight, and blood flow) and a need to account for these to improve assessment of renal metabolic clearance and its extrapolation to in vivo.

## Introduction

In vitro drug metabolism data obtained in enriched subcellular fractions such as microsomes or cytosol are commonly scaled using in vitro-in vivo extrapolation (IVIVE) to predict clearance in vivo (Houston and Galetin, 2008; Gertz et al., 2010; Nishimuta et al., 2014). This approach relies on robust estimates of physiologically relevant scaling factors, including the protein content of the subcellular fraction in the tissue of interest. Although liver scaling factors have been well characterized [e.g., microsomal (MPPGL) and cytosolic (CPPGL) protein per gram of liver] for human and several preclinical species (Houston, 1994; Barter et al., 2007; Smith et al., 2008; Cubitt et al., 2011), fewer data have been reported for extrahepatic tissues, such as the kidney (Gill et al., 2012; Scotcher et al., 2016a,b). Notably, data are completely lacking for microsomal (MPPGK) and cytosolic (CPPGK) protein per gram of kidney in preclinical species. Although several studies have reported microsomal protein yields for rat kidney, with some also reporting the corresponding data for liver, none of these reports stated clearly whether the protein recovery was estimated and accounted for (Jakobsson, 1974; Litterst et al., 1975; Sausen and Elfarra, 1990; Orellana et al., 2002). In addition, no data exist for CPPGK in humans, and an estimate of cytosolic protein content of liver is currently used as a surrogate for IVIVE (Säll et al., 2012; Nishimuta et al., 2014).

**Fig. 1.**
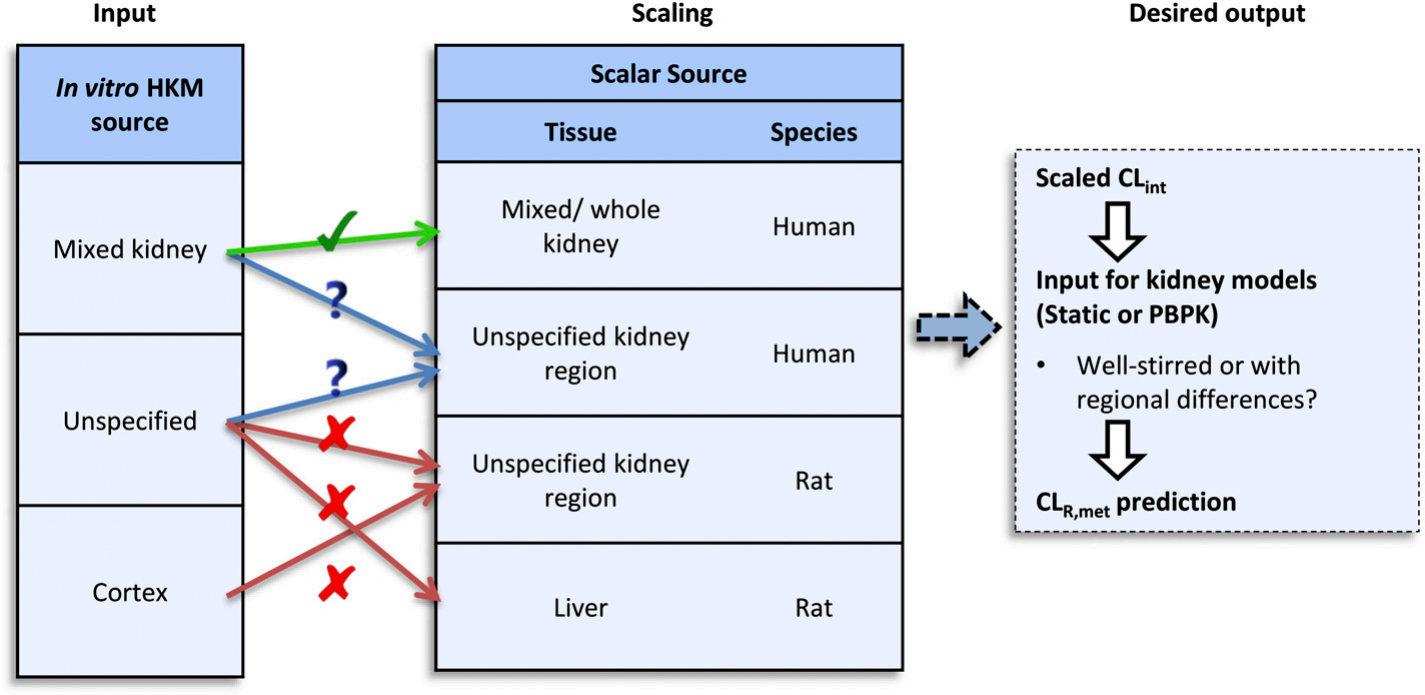
Comparison of kidney regions used to prepare HKMs for in vitro assays and different scaling factors currently used for extrapolation. Matrix-scalar combinations that have been used in the literature are annotated as being appropriate (green **✓**), inappropriate (red x) or ambiguous/ debatable (blue**?**). Typically, scaled intrinsic clearance (CL_int_) data are subsequently used as input into static or physiologically based kidney models for prediction of in vivo renal metabolic clearance (CL_R,met_). The assumptions of a particular kidney model (e.g., well-stirred or with regional/ cellular differences) will dictate the most appropriate matrix and scalar to use for in vitro metabolic data. Similarly, the availability of tissue for in vitro experiments (e.g., mixed kidney or cortex only) may limit the scaling factor and affect the selection of a kidney model. Adapted from Fig. 1 in Scotcher et al. (2016a) and references therein, licensed under CC BY 4.0 (http://creativecommons.org/licenses/by/4.0/).

The MPPGK values for humans range from 5.3 to 32.0 mg/g of kidney (data based on four literature reports, 23 donors, and different kidney regions), with weighted (by donor number) mean of 13.6 mg/g of kidney. Several differences in the designs of these studies are evident, for example, selection of microsomal protein marker and the region of kidney used (cortex, medulla, or mixed). It is therefore challenging to distinguish the contribution of true biologic variability and specific interstudy differences from the reported MPPGK values and to establish the most appropriate value to apply as a scaling factor, as summarized in Fig. 1. A value of 12.8 mg/g of kidney (based on five donors) is the most commonly used scalar for IVIVE of renal drug metabolism data (Scotcher et al., 2016a, b). The region of kidney used to obtain this commonly used scalar is unclear (Al-Jahdari et al., 2006). More recently, MPPGK data for mixed kidney have been reported (i.e., cortex and medulla) (Knights et al., 2016). Combining the data from these two studies resulted in a weighted mean MPPGK of 11.1 mg/g of kidney.

Kidney samples from mixed regions are also used for preparation of commercially available kidney microsomes (M. Farooq, XenoTech Ltd, Kansas City, KS). Use of mixed kidney microsomes for IVIVE of renal drug metabolism is supported by a recent study indicating that differences in uridine 5′-diphospho-glucuronosyltransferase (UGT) activity between microsomes prepared from the cortex or medulla are reduced when data are normalized for UGT protein abundance (Knights et al., 2016). This approach does not take into consideration other differences between the cortex and medulla, such as content of endoplasmic reticulum, tissue weight, and blood flows. More specifically, the cortex represents approximately 68% of kidney weight but receives about 80% of renal blood flow (Lerman et al., 1996; Vallée et al., 2000). Therefore, application of in vitro data obtained from mixed kidney microsomes in the well-stirred kidney model (often applied) may result in inaccurate IVIVE of renal metabolic clearance (CL_R,met_). This may be especially pertinent if data are generated to inform parameters of more mechanistic kidney physiologically based models that account for regional differences. Improved confidence in the IVIVE of metabolism by the kidney will increase the accuracy of predicting overall metabolic clearance, despite its generally smaller role compared with hepatic metabolism.

Measurement of microsomal or cytosolic protein contents requires the use of markers of the subcellular fraction of interest. Such markers are used in a quantitative manner to correct for any protein losses during centrifugation. Cytochrome P450 (CYP) content is frequently used as a marker for liver microsomal protein (Barter et al., 2008) but may not be suitable for the kidney as a result of the lower CYP content in this organ (Litterst et al., 1975; Song et al., 2015); therefore, alternative markers, such as glucose-6-phosphatase (G6Pase) and NADPH cytochrome c reductase activity are preferred (Scotcher et al., 2016b). The activities of glutathione-S-transferase (GST) and alcohol dehydrogenase have been reported in the literature for estimation of the cytosolic protein content of human liver (Cubitt et al., 2011); however, a more thorough assessment of the suitability of these enzymes as cytosolic protein markers for kidney is currently lacking.

The aim of this study was to characterize the microsomal and cytosolic protein content, as well as the functional activity, of kidney cortex samples from dogs and humans. CYP content and G6Pase activity were assessed as markers to measure microsomal protein recovery in dog kidney cortex and liver. In addition, the use of fresh and frozen tissue to prepare dog kidney cortex homogenates and microsomes and the impact on subsequent CYP content measurements and MPPGK estimates were assessed. Microsomal protein recovery in dog liver, kidney cortex, and intestine was compared using samples from the same animal donors. After method optimization in dogs, MPPGK and CPPGK were characterized for 38 human kidney cortex samples using G6Pase and GST activity as recovery markers, respectively. Impact of age and gender as covariates of MPPGK was investigated for 20 donors for which data were available. For the same subset, selected UGT polymorphisms and functional activity of prepared human kidney cortex microsomes were characterized using mycophenolic acid glucuronidation substrate depletion assay as activity marker. The mycophenolic acid unbound intrinsic clearance by glucuronidation obtained in human kidney cortex microsomes (CL_int,u,UGT,HKM_) was scaled by both historical MPPGK for the whole kidney and the newly acquired MPPGK data for the kidney cortex to assess the impact of revised scaling factors on predicted renal metabolic clearance.

## Materials and Methods

### Isolation of Microsomal Protein from Dog Kidney Cortex

#### Reagents.

Chemicals were purchased from Sigma-Aldrich (Gillingham, Dorset, UK) unless otherwise specified. Homogenization buffer was phosphate-buffered saline (PBS) with 0.5 mM EDTA, 5 mM histidine, and 0.25 M sucrose, pH 7.4. Storage buffer was 100 mM Trizma with 0.5 mM EDTA in deionized water, pH 7.4. CYP assay buffer was 25 mM potassium phosphate buffer, pH 7.4, with 1.5% w/v potassium chloride and 30% v/v glycerol (Fisher Scientific, Loughborough, UK). G6Pase assay buffer was 100 mM BIS-TRIS, pH 6.5. Taussky-Shorr color reagent (Taussky and Shorr, 1953) was 0.18 M ferrous sulfate heptahydrate, 1% w/v ammonium molybdate in 0.5 M sulfuric acid.

#### Sample Collection and Perfusion.

Kidneys and livers from 17 beagle dogs (4 males, 13 females) were obtained from necropsy at AstraZeneca (Alderley Park, Macclesfield, UK) according to institutional guidelines in compliance with national and regional legislation. The age and weights of the dogs ranged from 3.8 to 10.3 years and 19.7 to 20.5 kg, respectively. Liver weights were 315–709 g; kidney weights were 47–89 g. Livers were transferred to the laboratory in PBS on ice; kidneys were transferred in PBS containing 9 U/ml of heparin on ice. Kidneys were perfused with PBS containing 9 U/ml of heparin at 37°C at 8 ml/min for 15 min through the renal artery. All subsequent processes were performed on ice unless specified. Kidneys were cut in half and decapsulated, and each kidney half was blotted to remove excess liquid and weighed. Kidney halves from one kidney were frozen at −80°C, and the other kidney was used to prepare homogenate. Pieces of liver (∼10–20 g) were washed in PBS, weighed, and frozen at −80°C.

#### Dog Homogenate and Microsome Preparation.

Homogenization and centrifugation methods used for preparation of kidney microsomes vary but generally follow the same core strategy that involves an initial centrifugation of homogenate at around 9000–12,000 g to remove cellular debris and larger organelles, followed by ultra-centrifugation of the resulting supernatant at 100,000–110,000 g to obtain the microsomal protein pellet (Supplemental Fig. S1). The method applied in the current study, which was consistent with this core strategy, was based on the inhouse method developed for the intestine, with modifications to optimize homogenization of kidney (Hatley et al., 2017).

Frozen dog tissue samples, stored at −80°C, were rapidly thawed at 37°C, washed in PBS, blotted, and weighed. Kidney cortex (2.0–5.0 g) and liver (3.4–4.3 g) were minced with scissors and homogenized with 4 to 5 ml/g mince of homogenization buffer. Homogenization was initially with a rotor-stator homogenizer (Omni International, Kennesaw, GA) with a 10 mm × 95-mm probe. Bursts of 20 s with 30 s rest on ice were used until no intact pieces of tissue mince were apparent on visual assessment. The number of bursts for each sample depended on the starting weight of the minced tissue but required no more than eight bursts for kidney cortex and four bursts for liver. Samples were further homogenized using a VibraCell ultrasonic processor (Sonics & Materials, Inc., Newtown, CT) for two bursts of 10 s, separated with a 30 s resting period on ice to prevent excessive heat buildup. Homogenate was filtered through 170-*µ*m nylon mesh (Plastok Associates, Birkenhead, Merseyside, UK). Homogenate volumes were measured, and aliquots were stored on ice for analysis. Liver and kidney cortex homogenates were centrifuged at 9000*g* at 4°C for 15 min using an Optima LE-80K ultracentrifuge with a type 50.2Ti rotor (Beckman Coulter UK Ltd., High Wycombe, Buckinghamshire, UK). Supernatants were further centrifuged at 105,000*g* at 4°C for 70 min. Aliquots of the cytosol were retained. The microsomal pellet was resuspended in storage buffer using a handheld Potter-Elvehjem homogenizer. Samples were stored at −80°C.

#### Microsomal Protein Markers in Dog Samples.

Frozen samples were thawed rapidly at room temperature, and kept on ice until used (Pearce et al., 1996). Protein in homogenate, microsomes, and cytosol was determined using a microbicinchoninic acid protein assay kit (Pierce Biotechnology no. 23227; Pierce Biotechnology, Waltham, MA) according to the manufacturer’s instructions. Absorbance (562 nm) was measured with a Tecan Safire microplate reader with XFluor4 software (Reading, Berkshire, UK).

The CYP content of homogenate and microsomal samples was measured according to the dithionite difference spectroscopy method of Matsubara et al. (1976). Samples were diluted to 2 mg/ml in CYP assay buffer and bubbled (about one bubble/s) for 1 min with carbon monoxide. Then 1 ml of diluted samples were dispensed into each of two semi-microcuvettes (VWR, Radnor, PA), and baseline absorbance spectrum was measured (400–600 nm) using a UV-2401-PC dual-beam spectrophotometer with UVPC software (Shimadzu, Milton Keynes, Buckinghamshire, UK), and 10 *µ*l of freshly prepared sodium dithionite (200 mg/ml in CYP assay buffer) was added to the sample cuvette. The sample cuvette was inverted 4 times, left to stand for 4 min, and then the absorbance spectrum measured. CYP content (nmol/mg protein) was calculated using a molar extinction coefficient (A450–490) of 0.104 (Matsubara et al., 1976). Interassay variability of CYP content measurements was assessed by repeat measurements in four batches of homogenates and microsomes from three dogs (i.e., two batches prepared from the same animal).

Various endogenous contaminants, such as methemoglobin, cytochrome b_5_, and cytochrome oxidase, can potentially interfere with CYP content measurements in microsomal samples (Estabrook and Werringloer, 1978; Johannesen and DePierre, 1978; Burke and Orrenius, 1979). Furthermore, during preliminary experiments, broad absorbance peaks were observed at approximately 426 and 430 nm in homogenate and microsomes, respectively, which may have interfered with the A450 measurements and therefore affected CYP content measurement and MPPGK estimates. It was previously reported that this interference can be limited by chemically reducing the contaminants during the CYP content assay (Estabrook and Werringloer, 1978; Burke and Orrenius, 1979). In the current study, inclusion of 0.25 mM sodium ascorbate and 2.5 *µ*M phenazine ethosulfate, reported to reduce methemoglobin (Burke and Orrenius, 1979), did not cause substantial change in the dithionite difference spectra for dog kidney cortex microsomes, although a small shift in the 426-nm peak to 430 nm was noted (data not shown). Inclusion of NADH and sodium succinate, which are reported to reduce cytochrome b_5_ and cytochrome oxidase (Estabrook and Werringloer, 1978; Burke and Orrenius, 1979), in the CYP content assay buffer caused a change in the spectra of homogenate (∼400–420 nm) and microsomes (∼400–435 nm) (Supplemental Fig. S2). As no major change in baseline or peak at 450 nm was observed, neither the CYP measurements in homogenate and microsome samples nor the estimates of MPPGK were affected. Therefore, the sodium dithionite difference spectra assay as reported in the literature (Matsubara et al., 1976), that is, without modification of buffer constituents, was considered sufficient for estimation of MPPGK in dogs.

The G6Pase activity was measured in duplicate using a spectrophotometric method (Nordlie and Arion, 1966). Homogenate and microsomal protein and glucose-6-phosphate were preincubated separately in G6Pase assay buffer at 37°C for 10 min. Homogenate and microsomes (0.25 mg/ml) were added to the G6P (1 mM) to initiate the reaction, and an aliquot was immediately quenched (3:1) in 20% trichloroacetic acid on ice (*t* = 0 min). Additional aliquots were quenched at 5, 15, 30, and 60 min. After centrifugation at 4000 rpm for 10 min, samples and phosphorous standards were added in 1:1 ratio to Taussky-Shorr color reagent. Absorbance (660 nm) was measured with a Tecan Safire plate reader with XFluor4 software. Results were processed with Microsoft Excel. G6Pase activity was expressed as nanomolars of inorganic phosphate (P_i_) formed per min/mg protein based on the initial linear rate of P_i_ formation. Interassay variability for G6Pase activity was assessed by remeasurement of a single set of samples prepared from the kidneys of three different dogs in three separate assays.

### Isolation of Microsomal and Cytosolic Protein from Human Kidney Cortex

#### Reagents.

XenoTech mixed-gender pooled (13 donors) human whole/mixed kidney microsomes (lot. 1410120; 4-methylumbelliferone glucuronidation activity of 105 nmol per min/mg of protein) were obtained from Tebu-bio (Peterborough, Cambs, UK). Chemicals were purchased from Sigma-Aldrich (Gillingham, Dorset, UK) unless otherwise specified. Homogenization buffer was 25 mM Trizma, 0.5 mM EDTA, 5 mM histidine, 0.25 M sucrose, pH 7.4. Trizma was used as an alternative to PBS to reduce background signal in G6Pase assay. Storage buffer, G6Pase assay buffer, and Taussky-Shorr color reagent were prepared as described earlier herein for dogs. Mycophenolic acid glucuronidation assay buffer was 0.1 M phosphate buffer containing 3.45 mM MgCl_2_, 1.15 mM EDTA, and 115 *µ*M saccharic acid lactone (Kilford et al., 2009).

#### Sample Collection and Storage.

Normal human kidney cortex pieces from nephrectomy patients (*n* = 20), excised from the pole of the kidney contralateral to the tumor site, were obtained by the Biobank, Central Manchester University Hospitals NHS Foundation Trust (CMFT), UK. Kidney cortex pieces were snap-frozen within 1 h of excision and stored at −80°C. Informed consent was obtained from donors. Ethical approval for this research was obtained from National Research Ethics Service (NRES) Committee London, Camberwell St. Giles (REC ref. 13/LO/1896), with samples stored under Human Tissue Authority license.

Human kidney cortex homogenates (*n* = 18) were prepared from renal cortex from healthy kidneys unsuitable for transplant at Newcastle University, obtained under NRES ethical approval with informed consent from the donors. Homogenates from Newcastle University were stored at −80°C until used. No information on the time delay between organ isolation and storage was available.

#### Homogenate and Microsomal Preparation.

A single batch of homogenate and microsomes was prepared for each donor, with the exception of donor CMFT6, for which an initial batch was prepared for use in preliminary experiments; data generated during preliminary experiments were not included in analyses of the main data set. Frozen human kidney cortex samples were rapidly thawed at 37°C, washed in PBS, blotted dry, and weighed. Finely minced human kidney cortex samples (1.2–6.7 g) were homogenized with 4 to 5 ml/g of mince of homogenization buffer. Homogenization was initially with a rotor-stator homogenizer (Dremel UK, Middlesex, UK). Bursts of 20 s with 30 s rest on ice were used until no intact pieces of kidney cortex mince were apparent upon visual assessment. This typically required three to six bursts, depending on the starting weight of the kidney cortex mince. Samples were further homogenized using an Omni Ruptor 400 Ultrasonic homogenizer (Omni International, Kennesaw, GA) for two bursts of 10 s each, separated with a 30-s resting period on ice. Homogenate was filtered through 170-*µ*m nylon mesh (Plastok Associates). Homogenates from Newcastle University were thawed rapidly at 37°C and then kept on ice until use. Total kidney cortex homogenate volumes were measured, and aliquots were stored on ice for analysis.

Human kidney cortex homogenates were centrifuged at 9000*g* at 4°C for 15 min using an Optima TLX-120 Ultracentrifuge with an MLA-80 rotor (Beckman Coulter UK Ltd). After removing aliquots for analysis (1 to 2 ml, stored on ice), 9000*g* supernatants (S9) were further centrifuged at 105,000*g* at 4°C for 70 min. Aliquots of the cytosol were stored on ice for analysis. The microsomal pellet was resuspended in storage buffer using a vortex mixer and pipette. Aliquots were taken for protein content analysis; remaining microsomal samples were stored at −80°C.

#### Microsomal and Cytosolic Protein Markers in Human Samples.

On the day of microsomal preparation, protein content in homogenate, S9, microsomes, and cytosol was determined in triplicate using a Micro Bicinchoninic Acid Protein Assay Kit (Pierce Biotechnology no. 23227) according to the manufacturer’s instructions. Absorbance (562 nm) was measured with a SpectraMax 190 plate reader (Molecular Devices, Sunnyvale, CA), with BSA used as calibration standard. All activity assays were performed on samples that had undergone four or fewer freeze-thaw cycles. G6Pase activity was measured in duplicate using the spectrophotometric method described earlier herein for the dog samples; absorbance (660 nm) was measured with a SpectraMax 190 plate reader. Interassay variability was assessed using four batches of human kidney cortex homogenate and microsomes from three kidney cortex samples, for which G6Pase activity was measured twice. Interbatch variability was assessed through preparation of two batches of homogenate and microsomes (donor CMFT6) on different days. Interbatch and interassay variability were compared by measuring G6Pase activities for each batch in two separate assays, with one of these assays common for both batches.

GST activity was measured in human kidney cortex homogenate, microsomes, and cytosol samples using an assay kit (Sigma no. CS0410) according to the manufacturer’s instructions with the following modification: samples were initially prepared in 0.1 M sodium phosphate buffer, pH 6.5, with 1% Triton X-100 (Ji et al., 2002) owing to inadequate volume of sample buffer provided with the assay kit. GST activity was measured using protein concentrations of 10 *µ*g/ml (determined after preliminary optimization experiments using rat kidney samples), with substrate concentrations of 100 and 200 *µ*M for 1-chloro-2,4-dinitrobenzene and l-glutathione, respectively. Absorbance (340 nm) was measured at appropriate time points up to 10 min using a SpectraMax 190 plate reader. Results were processed with Microsoft Excel. GST activity was expressed as nmol/min/mg protein based on the initial linear rate of ∆A340, using an extinction coefficient (∆A_340_) of 9.6 mM^−1^ cm^−1^ for 1-chloro-2,4-dinitrobenzene conjugate.

### Estimation of Microsomal and Cytosolic Protein Contents of Tissues

Various parameters (Table 1), including yields of total protein and microsomal marker in subcellular fractions from a microsomal preparation, as well as the recovery factor of the microsomal protein, were calculated (eq. 1–4). This approach allowed correction for the removal of material as aliquots of homogenate and S9 before differential centrifugation steps when calculating the theoretical yield of the protein marker (eq. 2). The latter represents the marker activity/content if there was a complete recovery of the marker that was present in the homogenate (eq. 2). Actual (eq. 3) and theoretical yield of the marker activity/content in the microsomal fraction obtained from the homogenate were used to calculate MPPGK (eq. 5). In addition, a microsomal or cytosolic protein enrichment factor was calculated based on the marker activity/content of the subcellular fraction relative to that of the homogenate (eq. 6):

**TABLE 1:** Parameters used in calculation of MPPGK and CPPGK from human and dog kidney cortex samples

Parameter*^a^*	Description	Units
Abs_Prot_x_	Absolute protein yield in homogenate or subfraction (*x*)	mg
[Prot]_x_	Protein concentration of homogenate or subfraction (*x*)	mg/ml
V_x, total_	Volume of homogenate or subfraction (*x*), before aliquots are taken for analysis where applicable	ml
V_x, aliquot_	Volume of homogenate or subfraction aliquot taken for analysis	ml
Marker_x_	Activity or content of subcellular protein marker in homogenate, microsome, or cytosol (*x*)	nmol/mg protein (CYP)
		nmol/min/mg protein (G6Pase)
		nmol/min/mg protein (GST)
W_Kid_	Weight of starting kidney tissue mince	g
Yield_Marker, theor_	Theoretical yield of subcellular protein marker from homogenate, accounting for aliquot removal	nmol (CYP)
		nmol/min (G6Pase)
		nmol/min (GST)
Yield_Marker, actual_	Actual yield of subcellular protein marker from homogenate	nmol (CYP)
		nmol/min (G6Pase)
		nmol/min (GST)
Recovery_X_	Percent recovery	%
Enrichment_x_	Enrichment factor of subcellular protein (*x*)	
Mic_ Prot_Hom_	Amount of microsomal protein in the homogenate, based on starting tissue weight and the MPPGK.	mg
Mic_GST_Hom_	Activity of GST in the homogenate attributable to microsomal isoform(s)	nmol/min
Yield_GST,theor,corrected_	Theoretical cytosolic GST activity yield. The GST activity yield in the homogenate that was attributed to the cytosolic fraction (i.e., corrected for the microsomal GST activity)	nmol/min
S9_contribution_Hom_	Theoretical % contribution of the microsomal protein and cytosolic protein (i.e., S9 fraction) to overall protein in homogenate	%

^*a*^ Where *x* represents either homogenate (Hom), 9000*g* supernatant (S9), or microsomes (Mic). Equations are stated in the *Materials and Methods* (eq. 1–12).

(1)
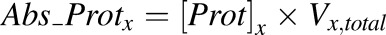
(2)
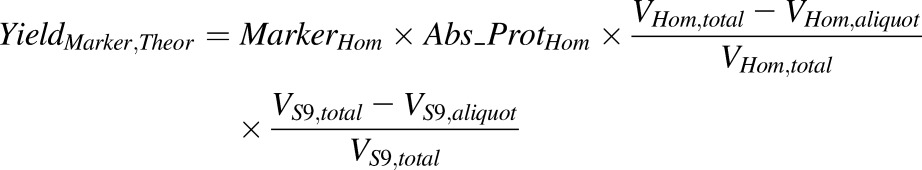
(3)
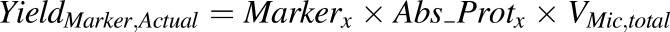
(4)
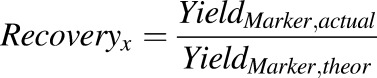
(5)
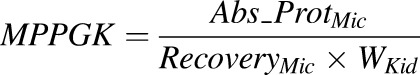
(6)
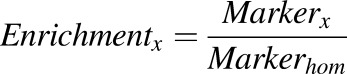
The preceding equations are applicable for calculation of the cytosolic protein recovery and CPPGK in conjunction with appropriate cytosolic protein markers. GST can be considered a cytosolic marker, with a limitation that some GSTs are also found in the endoplasmic reticulum component of the microsomal fraction (Hayes and Pulford, 1995; Song et al., 2015). In an exploratory assay, substantial GST activity was noted in human kidney cortex microsomes, suggesting that GST activity in human kidney cortex homogenate was attributable to both cytosolic and microsomal isoforms (Supplemental Fig. S3). Therefore, MPPGK for each human kidney donor, estimated using G6Pase activity as microsomal protein marker, was used to account for the GST activity attributable to the microsomal GST in each human kidney cortex homogenate (eq. 7–9). This involved calculating the total microsomal protein and then the microsomal GST activity in the homogenate, which was subtracted from the theoretical GST yield (calculated using eq. 2). This corrected theoretical GST activity yield in homogenate was compared with the actual GST activity yield in the cytosolic fraction (eq. 3) to account for cytosolic protein losses during the fractionation procedure and subsequently CPPGK (eq. 10 and 11).

To ensure that the estimates of MPPGK and CPPGK were physiologically feasible, their combined value was compared with the amount of homogenate protein obtained per gram of kidney cortex for each donor. The combined value should reflect the S9 protein content per gram of kidney cortex. Therefore, the value calculated was expressed as the percent contribution of the S9 fraction to the overall protein in the homogenate (eq. 12):

(7)
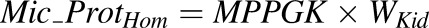


(8)
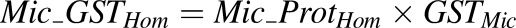


(9)
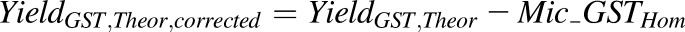


(10)
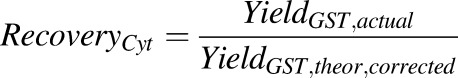


(11)
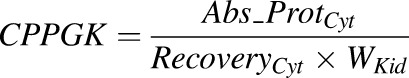


(12)
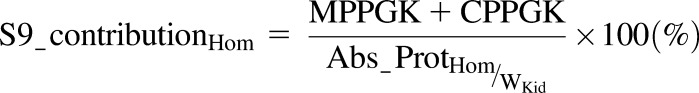


### Mycophenolic Acid Glucuronidation Depletion Assay in Human Kidney Microsomes

Mycophenolic acid was selected as a clinically relevant marker to assess the metabolic activity of the prepared human kidney cortex microsomes, and investigate the variability of UGT activity within the kidney cortex samples. Mycophenolic acid has previously been shown to undergo glucuronidation in vitro in human liver, intestine, and kidney microsomes (Picard et al., 2005; Cubitt et al., 2009; Gill et al., 2012), with UGT1A9 identified as the major enzyme involved in its renal metabolism and UGT2B7 having a lesser role (Picard et al., 2005). Microsomal glucuronidation substrate depletion intrinsic clearance assays were performed for a subset of 20 donors (CMFT) using a method previously reported (Gill et al., 2012), including a no-cofactor control. The mycophenolic acid reactions were performed at a substrate concentration of 1 *µ*M, which was expected to be under linear conditions considering the reported *K*_m_ values for UGT1A9 and UGT2B7 (Bernard and Guillemette, 2004; Picard et al., 2005). Because of low availability of microsomal protein, only one replicate for each donor was performed; each assay was done in triplicate. The assay was also performed in XenoTech pooled human kidney microsomes (13 donors, mixed gender). Human kidney microsomes (0.25 mg/ml) were activated by preincubation with 50 *µ*g/mg protein alamethicin in assay buffer for 15 min on ice. Mycophenolic acid was preincubated with alamethicin-activated microsomes and bovine serum albumin (BSA; assay concentration 1%) for 5 min in assay buffer at 37°C shaking at 900 rpm (Eppendorf thermomixer; Hamburg, Germany)). Reaction was initiated by the addition of uridine-diphosphate-glucuronic acid at a final assay concentration of 5 mM. After incubation at 37°C with shaking at 900 rpm, aliquots of the incubation mixture were quenched in two volumes of ice-cold acetonitrile containing 1 *µ*M warfarin (internal standard) at eight time points between 0 and 60 min inclusive. Minimal depletion of mycophenolic acid was observed after 60 min at 0.25 mg/ml for donor CMFT1; therefore, a modified assay, with a protein concentration of 0.5 mg/ml and time points extended to 90 min, was used for this donor. Quenched samples were stored at −20°C for at least 1 h and then centrifuged at 9000 rpm for 20 min. Aliquots of supernatant were analyzed by liquid chromatography-mass spectrometry (LC-MS/MS) for mycophenolic acid concentration using matrix-matched calibration standards (0–5 *µ*M). To preserve individual donor human kidney cortex microsome samples, XenoTech pooled human kidney microsomes were used for preparing calibration standards.

LC-MS/MS analysis was performed using an Agilent 1100 HPLC system (Stockport, Cheshire, UK) coupled to a Micromass Quattro Ultima triple quadruple mass spectrometer (Waters, Elstree, Hertfordshire, UK). LC was performed using a Luna C18 (3 *µ*, 50 × 4.6 mm) column (Phenomenex, Torrance, CA) with appropriate elution gradient (Supplemental Table S1) and a flow rate of 1 ml/min. The retention times of mycophenolic acid and warfarin were 4.21 and 4.49 min, respectively. For MS, source temperature, desolvation temperature, desolvation gas flow rate, cone gas flow rate, and capillary voltage were 125°C, 350°C, 600 l/h, 50 l/h, and 3.5 kV, respectively. Selective reaction monitoring of mycophenolic acid and warfarin with negative electrospray ionization was performed; transitions of precursor to product ions (*m/z*) were 318.90→191.10 for mycophenolic acid and 306.90→161.05 for warfarin. Cone voltage and collision voltage were 90 V and 25 eV for mycophenolic acid and 130 V and 19 eV for warfarin, respectively.

### Genotyping of Selected Polymorphisms in UGT1A8, 1A9, and 2B7

Genotyping of 20 human kidney cortex samples for selected single-nucleotide polymorphisms (SNPs) genes encoding the UGT1A8 (rs17863762), UGT1A9 (rs17868320, rs2741045, rs6714486, rs72551330, rs2741046), and UGT2B7 (rs7438135) enzymes was performed by NewGene (Newcastle upon Tyne, UK). These SNPs were selected on the basis of clinical data indicating that they are associated with interindividual variability in pharmacokinetic and pharmacodynamic endpoints of mycophenolic acid (Picard et al., 2005; Prausa et al., 2009; Fukuda et al., 2012). Briefly, after DNA extraction from tissue using a Promega Maxwell automation platform, polymerase chain reaction, and extension reaction, analysis was performed on the Agena MassARRAY4 platform. Each sample was run in duplicate.

### Prediction of In Vivo Mycophenolic Acid Glucuronidation Clearance

Human kidney cortex microsomal intrinsic clearance (CL_int,UGT,HKM_; *µ*l/min/mg of microsomal protein) for mycophenolic acid was calculated from the elimination rate constant (*k*; min^−1^) and the microsomal protein concentration of the incubation (mg/ml) using eq. 13; *k* was calculated from the slope of the linear correlation of the natural log-fraction remaining (average of triplicate incubations at each time point) versus time. In vitro CL_int,UGT,HKM_ data for each donor were corrected for the fraction unbound in the incubation (*f*_u,inc_; 0.18 at all microsomal protein concentrations, obtained in the presence of 1% BSA, as previously reported) (Gill et al., 2012) to calculate the unbound intrinsic clearance (CL_int,u,UGT,HKM_). The CL_int,u,UGT,HKM_ data were scaled using MPPGK and average kidney weight of 4.5 g/kg of body weight. Prediction of in vivo mycophenolic acid renal glucuronidation clearance (CL_R,met,UGT_) was done using the well stirred kidney model (eq. 14), fraction unbound in plasma (*f*_u,p_) and blood-to-plasma concentration ratio (*R*_B_) of 0.01 and 0.6, respectively (Gill et al., 2012).

The IVIVE of mycophenolic acid CL_R,met,UGT_ was performed using two different scenarios for scaling factors, as summarized in Table 2. An MPPGK of 11.1 mg/g kidney was applied in Scenario 1; this value was calculated as the weighted (by donor number) mean of literature values reported by studies that used mixed kidney (i.e., cortex and medulla) or unspecified region (Al-Jahdari et al., 2006; Knights et al., 2016). In Scenario 2, the CL_int,u,UGT,HKM_ values for each donor were scaled by the corresponding MPPGK value obtained for kidney cortex in the current study. Prediction of in vivo metabolic clearance also requires information on organ weight and blood flow; for Scenario 1, whole kidney weight and renal blood flow (*Q*_R_) were used, whereas cortex weight and cortical blood flow were used in Scenario 2 (68% and 80% of the respective values for the whole kidney (Lerman et al., 1996; Vallée et al., 2000) (Table 2).

**TABLE 2:** Physiologic values used for CL_R,met,UGT_ predictions using IVIVE in different scenarios

Parameter (U)	Scenario 1 (Whole Kidney)	Scenario 2 (Kidney Cortex)
MPPGK (mg/g kidney)	11.1*^a^*	Donor specific*^b^*
Kidney weight (g/kg body weight)	4.5	3.1
Renal blood flow (ml/min/kg body weight)	16.4	13.2

^*a*^ Weighted (by number of donors) mean of values reported for microsomes prepared from mixed kidney or unspecified region (Al-Jahdari et al., 2006; Knights et al., 2016).

^*b*^ Fig. 5 and (Supplemental Table S3).

Predicted overall mycophenolic acid glucuronidation clearance rates were calculated as the sum of the renal (eq. 14) and hepatic (CL_h,met,UGT_) glucuronidation clearances (eq. 15). Analogous to renal metabolism, CL_h,met,UGT_ was calculated with the well stirred liver model, using scaled CL_int,u,UGT,HLM_ of 9.32 ml/min/g liver, obtained under the same BSA conditions in vitro, as reported in Gill et al. (2012). MPPGL of 40 mg/g of liver, liver weight of 21.4 g/kg of body weight, and hepatic blood flow (Q_h_) of 20.7 ml/min/kg were used, as previously reported (Gill et al., 2012). Observed mycophenolic acid glucuronidation clearance (CL_UGT_) of 3.97 ml/min/kg (Gill et al., 2012) was used to assess the predictive performance of the IVIVE. This value is based on a plasma i.v. clearance of 2.49 ml/min/kg corrected for the renal excretion (0.01 ml/min/kg) and the fraction metabolized by UGT (f_m,UGT_ of 0.95, obtained from urinary excretion data):

(13)
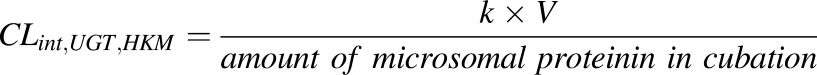


(14)
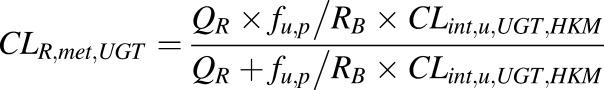


(15)
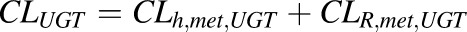


### Data Analysis

CYP content and microsomal protein per gram of intestine (MPPGI) data for 14 dog intestinal samples were provided by Dr Oliver Hatley (manuscript in preparation). These data were obtained from different regions of the intestine, with each region being defined as one sixth of the entire intestine by length. The initial three regions were defined as proximal 1, 2, and 3; the final region was defined as distal.

Average (mean) values were calculated, with variability estimated using the coefficient of variation (CV; %). Interassay variability (%) was estimated as the average between-assay CV for each set of samples. Data were analyzed using MS Excel. Student’s *t* test (paired, two-tailed) was used to statistically compare means; *P* < 0.05 was considered significant. The unpaired *t* test was used for comparison of CYP content in homogenates prepared from fresh and frozen kidney cortex owing to differences in the number of samples in each group.

## Results

### Characterization and Optimization of Protein Marker Assays

In the initial phase of the study, the validity of three different markers was investigated, together with assessment of assay reproducibility.

#### CYP Content Assay.

Compared with the liver, 450 nm absorbance signal in the sodium dithionite difference spectra was generally weak in kidney cortex but sufficient for quantification. On average, the interassay variability of CYP content was 10% and 5% for homogenate and microsomes, respectively, and 14% for the calculated microsomal protein enrichment factor. Based on data from one dog, for which two separate batches of microsomes were prepared, the interassay variability in CYP content measurement was similar to the apparent interbatch variability (Supplemental Fig. S4). This trend was also noted for the calculated CYP content enrichment factor (approx. 12% variability for interbatch and interassay).

#### G6Pase Activity Assay.

Dog kidney cortex G6Pase activity appeared to be linear with respect to protein concentration in both homogenate and microsomes, but it was not directly proportional (i.e., intercept ≠ 0) (Supplemental Fig. S5). Activity could not be reliably quantified at the lower protein concentrations (≤0.1 mg/ml) for homogenate. The resultant microsomal protein recovery factors calculated for each assay protein concentration did not appear to show protein dependency. Therefore, G6Pase activity was considered a suitable marker to estimate microsomal protein losses. The average interassay variability (CV) of G6Pase activity was 20.6% and 19.8% for homogenate and microsomes, respectively, whereas G6Pase activity enrichment factor interassay variability was 14%.

In human kidney cortex, the interassay variability of G6Pase assay appeared to be greater than the interbatch variability (Supplemental Fig. S6). The average interassay variability in G6Pase activity was 15% and 19% for homogenate and microsomes, respectively, which resulted in an average interassay variability of 18% for the calculated G6Pase activity enrichment factor (range, 3%–39%).

#### GST Activity Assay.

GST activity was nonlinear with respect to protein concentration in both rat kidney homogenate and cytosol (Supplemental Fig. S7). GST activity could be reliably quantified at the lower protein concentrations (≤5 *µ*g/ml), albeit with lower reproducibility in homogenate. There was low interassay variability at the protein concentration selected for the final assay (10 *µ*g/ml). Assay protein concentration did not appear to affect the apparent enrichment factor (Supplemental Fig. S7). Therefore, GST activity was considered a suitable marker for cytosolic protein.

### Estimation of Microsomal Protein Content in Dog Kidney Cortex and Liver and Comparison with Intestine

Liver and kidney cortex samples were obtained from a total of 17 dogs. Average CYP content in dog kidney cortex homogenate prepared from frozen kidney tissue was 0.056 nmol/mg protein (*n* = 17), which was significantly lower (*P* < 0.05) than that in homogenate prepared from fresh kidney cortex tissue (0.086 nmol/mg protein; *n* = 14) (Table 3). Both CYP content and G6Pase activity were statistically significantly lower (*P* < 0.05) in dog kidney cortex compared with corresponding livers (data were available only for frozen tissue samples). Mean CYP content for dog kidney cortex microsomes was more than 3-fold greater than for intestinal microsomes (samples were available from fresh tissue only). No trends were apparent in the CYP content or G6Pase activity between the liver and kidney cortex, based on visual assessment of the data.

**TABLE 3:** CYP content, G6Pase activity, and MPPG measured in homogenate and microsomal samples prepared from fresh dog kidney cortex, frozen dog kidney cortex, and frozen dog liver Average values are presented, with CVs in parentheses. G6Pase activity was not measured in samples prepared from fresh dog kidney cortex. Data for individual dogs are presented (Supplemental Table S2).

		CYP Content (nmol/mg Protein)		G6Pase Activity (nmol/min/mg Protein)		MPPG (mg/g Tissue)	
		Homogenate	Microsomes	Homogenate	Microsomes	CYP content	G6Pase activity
Fresh tissue (*n* = 14)	Dog kidney cortex	0.086 (24%)	0.205 (23%)	Not measured	Not measured	43.1 (22%)	Not measured
	Dog intestine*^a^*	Data not available	0.059 (27%)	Not measured	Not measured	6.5 (61%)	Not measured
Frozen tissue (*n* = 17)	Dog kidney cortex	0.056 (16%)	0.230 (15%)	19.9 (16%)	62.1 (16%)	33.9 (18%)	44.0 (16%)
	Dog liver	0.113 (19%)	0.665 (20%)	23.8 (15%)	91.2 (18%)	41.1 (12%)	63.6 (18%)

^*a*^ Data for dog intestine were provided by Dr Oliver Hatley (manuscript in preparation) and represent data pooled from several intestinal regions.

**Fig. 2.**
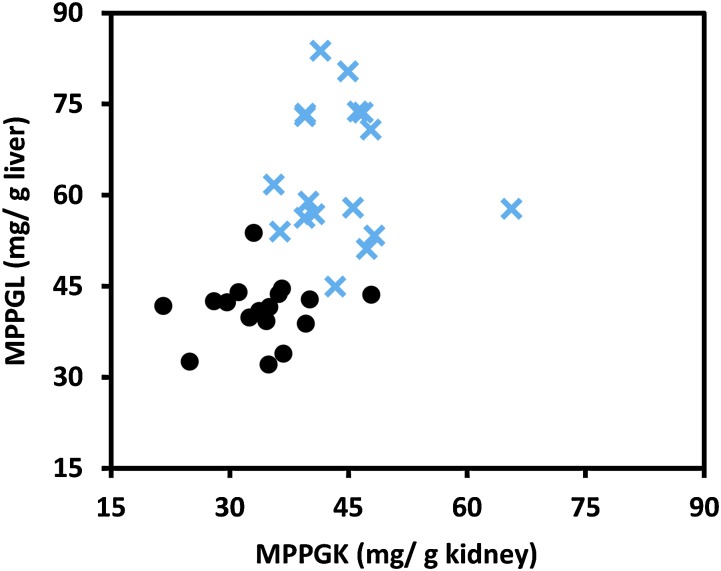
Comparison of MPPGK and MPPGL in dogs (*n* = 17 dogs) using either CYP content (black circle) or G6Pase activity (blue cross) as the microsomal protein marker. Each point represents microsomal scalar measured using a single batch of homogenates and microsomes from a single dog.

**Fig. 3.**
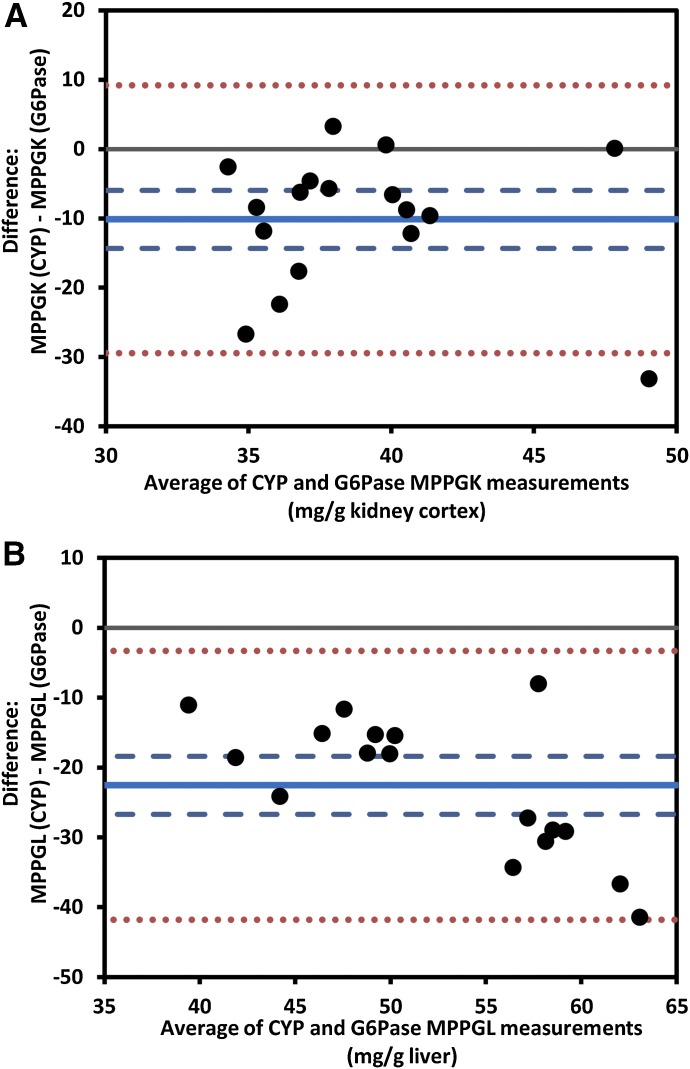
Bland-Altman plots: difference in MPPG measured using CYP content versus G6Pase activity as microsomal protein marker. Points on graphs represent measurements made in kidney cortex (**A**) or liver (B) microsome and homogenate samples. Blue lines represent mean (solid) and 95% confidence interval of mean (dashed) difference between MPPGs. Red dotted lines represent 95% limits of agreement. Thin black lines represent line of unity.

Mean MPPGK in dog kidney cortex was 43.1 mg/g kidney cortex when CYP content was used as microsomal protein marker and samples were prepared from fresh kidney cortex (Table 3); individual values ranged from 27.4 to 58.6 mg/g kidney cortex (Supplemental Table S2). This was on average 27% higher than the corresponding value when samples were prepared from frozen kidney cortex. MPPGK was on average 18% or 31% lower than MPPGL when CYP content or G6Pase activity was used as microsomal protein marker, respectively (Table 3). This difference varied between dogs, but no apparent correlation was found in MPPGK and MPPGL (Fig. 2). Both MPPGL and MPPGK were consistently greater than MPPGI for all regions of intestine studied, with no trends apparent, either when considering data for each region separately or data for all intestinal regions collectively. No clear trends between either MPPGL or MPPGK and factors such as age or dog weight were apparent (data not shown). Dog microsomal protein content was lower when using CYP content than when using G6Pase activity as microsomal marker, by 23% for MPPGK and 35% for MPPGL (Table 3). Bland-Altman plots show that the 95% confidence intervals for the mean difference between the markers do not overlap with the line of unity (difference = 0), suggesting systematic bias (Fig. 3).

### Estimation of MPPGK and CPPGK in Human Kidney Cortex

**Fig. 4.**
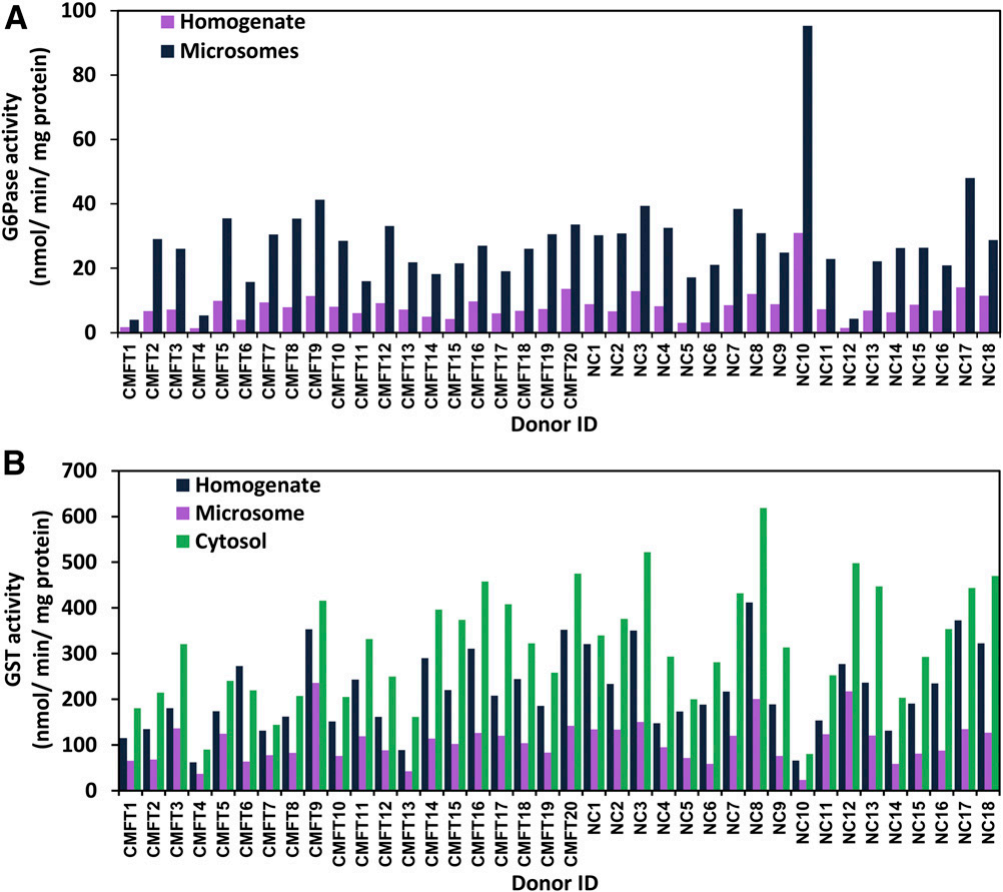
Marker activities measured in 38 human kidney cortex samples. (A) G6Pase activity in homogenate and microsomes. (B) GST activity homogenate, microsomes, and cytosols. CMFT number and NC number indicate samples acquired from the CMFT Biobank or Newcastle University, respectively. Each bar typically represent *n* = 1 measurements per donor, although for some samples bars represent the average of *n* = 2 measurements. Individual values are listed (Supplemental Table S3).

Average G6Pase activities of human kidney cortex homogenate and microsomes were 8.1 and 27.9 nmol/min/mg protein (*n* = 38 kidney cortex samples), with CVs of 61% and 53%, respectively (Fig. 4A). The G6Pase activities were higher in samples obtained from Newcastle University (9.2 and 31.1 nmol/min/mg protein in homogenate and microsomes, respectively; *n* = 18) compared with those obtained from the CMFT Biobank (7.1 and 24.9 nmol/min/mg protein in homogenate and microsomes, respectively; *n* = 20). Average GST activities of human kidney cortex homogenate, microsomes, and cytosol were 217, 106, and 318 nmol/min/mg protein, respectively (*n* = 38); CVs for those samples were between 40% and 44% (Fig. 4B). Analogous to G6Pase, GST activities were higher in samples obtained from Newcastle University (234, 112, and 357 nmol/min/mg protein in homogenate, microsomes, and cytosol; *n* = 18) compared with those obtained from CMFT Biobank (202, 100, and 284 nmol/min/mg protein in homogenate, microsomes, and cytosol, respectively; *n* = 20).

**Fig. 5.**
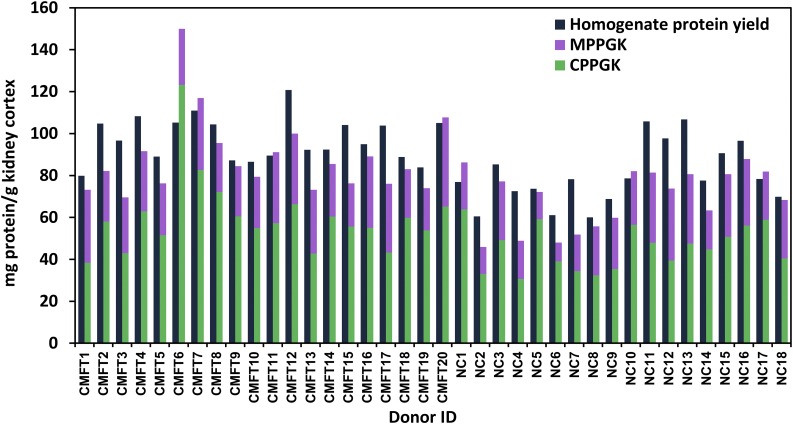
MPPGK and CPPGK protein content of kidney cortex and homogenate protein yields in 38 human kidney cortex samples. Combined value of MPPGK and CPPGK in each donor represents the estimated S9 protein per gram of kidney cortex; this value should not exceed the homogenate protein yield to be physiologically plausible. CMFT number and NC number indicate samples acquired from the CMFT Biobank or Newcastle University, respectively. Each bar represents *n* = 1 batch of homogenate/microsomes/cytosol per donor. Individual values are listed (Supplemental Table S3).

Average MPPGK in humans obtained from all 38 samples was 26.2 mg of protein/g kidney cortex, with a CV of 27% (Fig. 5 and Table 4). Microsomal GST activity, scaled using MPPGK to units of nmol/min/g kidney cortex, represented on average 14.5% of the GST activity yield in human kidney cortex homogenate. After correction for activity attributable to microsomal GST isoform(s) in the homogenate, average human CPPGK was 53.3 mg protein/g kidney cortex, with 31% CV (Fig. 5 and Table 4). There was no apparent trend between MPPGK and CPPGK (Supplemental Fig. S8). The average S9 protein per gram of kidney cortex (i.e., the sum of MPPGK and CPPGK) was 79.5 mg protein/g kidney cortex (*n* = 38). Theoretical contribution of the S9 protein to the protein content of homogenate was 89% on average, although the value exceeded 100% for seven of 38 samples (Fig. 5). Based on the subset of 20 donors for whom demographic data were available, no trends between human MPPGK or CPPGK and factors, such as age, gender, and weight, were found (not shown). MPPGK and CPPGK of samples from CMFT Biobank were each significantly greater than the values obtained from samples from Newcastle University (*P* < 0.05; two-tailed *t* test). Observed MPPGK variability for CMFT Biobank samples was one third lower than Newcastle University samples (Table 4).

**TABLE 4:** MPPGK, CPPGK, and S9PPGK for samples prepared from frozen human kidney. Data for individual donors are presented (Supplemental Table S3)

	MPPGK (mg Protein/g Kidney Cortex)	CPPGK (mg Protein/g kidney Cortex)	S9PPGK (mg Protein/g Kidney Cortex)
All donors (*n* = 38)			
Average	26.2	53.3	79.5
CV (%)	27	31	24
Range	9.0–42.6	30.6–123.2	45.9–149.9
CMFT donors only (*n* = 20)			
Average	28.4	60.3	88.7
CV (%)	21	30	21
Range	20.2–42.6	38.4–123.2	69.6–149.9
NC donors only (*n* = 18)			
Average	23.7	45.5	69.2
CV (%)	32	23	21
Range	9.0–34.3	30.6–63.8	45.9–87.8

### In Vitro Glucuronidation of Mycophenolic Acid by Human Kidney Cortex Microsomes and IVIVE

**Fig. 6.**
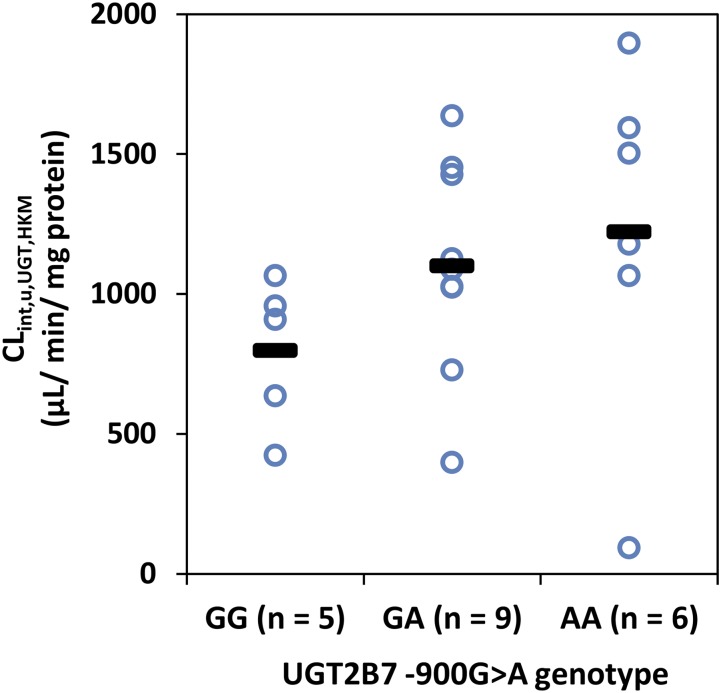
Individual (blue open circle) and mean (black line) mycophenolic acid CL_int,u,UGT,HKM_ (*µ*l/min/mg protein) for donors with different allelic variants for the −900G > A SNP in the UGT2B7 gene (rs7438135).

Mycophenolic acid CL_int,u,UGT,HKM_ was measured in 20 CMFT Biobank individual human kidney cortex microsomes and XenoTech pooled kidney microsomes (Supplemental Fig. S9). Average CL_int,u,UGT,HKM_ in the 20 donors was 1061 *µ*l/min/mg microsomal protein, with 43% CV and range of 93–1896 *µ*l/min/mg microsomal protein for donor CMFT1 and CMFT5, respectively. The average value was approximately 2-fold lower compared with mycophenolic acid CL_int,u,UGT,HKM_ obtained in the commercially sourced pooled kidney microsomes in the current study (1843 *µ*l/min/mg protein). No depletion of mycophenolic acid was observed in the no-cofactor control for any of the donors investigated. A positive correlation between mycophenolic acid CL_int,u,UGT,HKM_ and G6Pase activity was noted (Supplemental Fig. S10). A weak trend between mycophenolic acid CL_int,u,UGT,HKM_ and UGT2B7 genotype −900G > A (rs7438135) was noted (AA > GA > GG (Fig. 6); the low number of donors relative to the number of polymorphisms tested precluded statistical assessment of this trend. This trend was reflected in the predicted CL_UGT_, as six of seven of the donors with predicted/observed CL_UGT_ < 1.0 (Scenario 2) had the GG or GA genotype. No other trends between genotype and mycophenolic acid CL_int,u,UGT,HKM_ were apparent for the polymorphisms investigated (Supplemental Table S3).

**Fig. 7.**
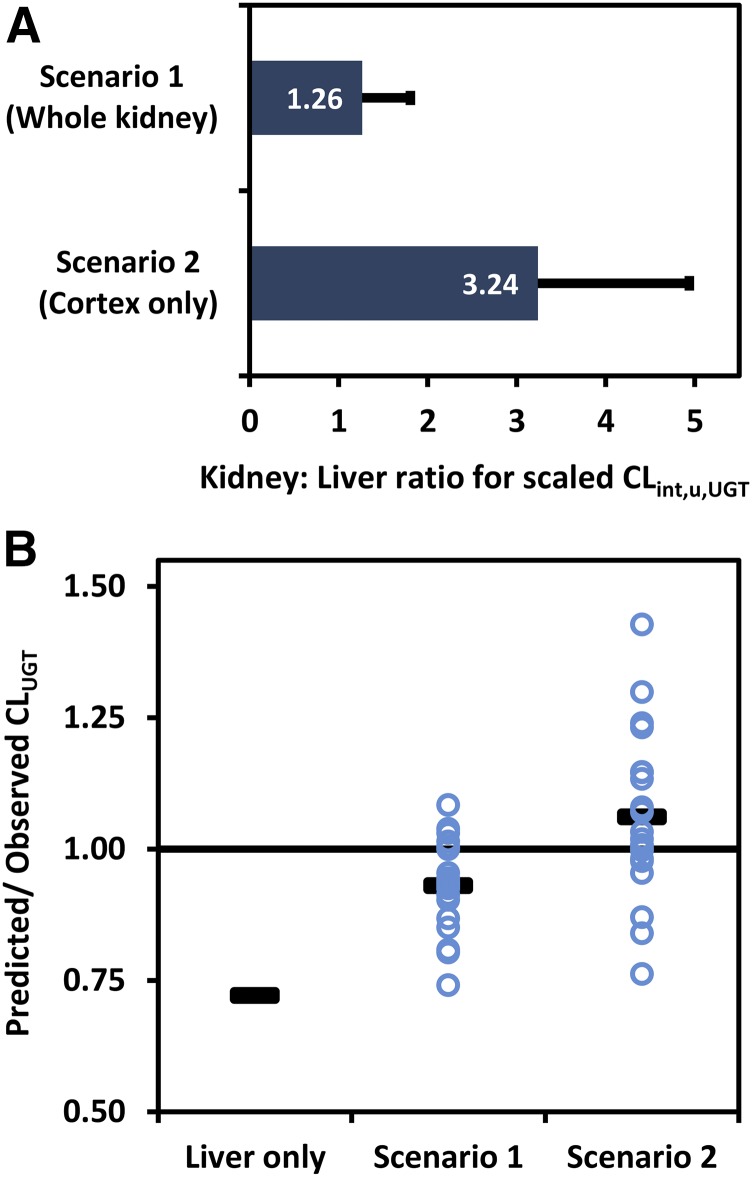
IVIVE of mycophenolic acid clearance under two different scenarios. MPPGK, kidney weight, and blood flow parameters used for scaling and in the well stirred kidney model represented either the whole kidney (Scenario 1) or kidney cortex (Scenario 2); details are listed in Table 5. (A) Kidney: liver ratios of scaled mycophenolic acid CL_int,u,UGT_ (ml/min/g tissue). Bars represent mean values; error bars represent the standard deviation. (B) Prediction accuracy of mycophenolic acid CL_UGT_, considering either the hepatic glucuronidation alone or the sum of the hepatic and renal glucuronidation clearances. The contribution of renal glucuronidation was predicted using two scenarios. Individual (blue open circle) and mean (black line) data are shown (*n* = 20). Solid horizontal line represents line of unity.

Scaled mycophenolic acid CL_int,u,UGT,HKM_ (per gram of organ weight) was on average 2.6-fold greater when the donor-specific MPPGK values measured using cortex tissue in the current study were applied (i.e., Scenario 2) than when the MPPGK value calculated for whole kidney was used (i.e., Scenario 1) (Table 5). These differences were reflected in the assessment of the importance of renal glucuronidation relative to liver, i.e., the kidney:liver ratios for CL_int,u,UGT_ (calculated using published data for liver, obtained using comparable in vitro assay conditions to the current study, i.e., 1% BSA) (Gill et al., 2012) (Fig. 7A).

**TABLE 5:** Comparison of scaled mycophenolic acid CL_int,u,UGT,HKM_ and predicted CL_UGT_ in scenarios that take different assumptions for physiologic parameters (see Table 2) Mean values from 20 individual human kidney cortex microsomes are shown, with CVs in parentheses. Data for individual donors are listed (Supplemental Table S3).

	Scenario 1	Scenario 2
CL_int,u,UGT,HKM_ (*µ*l/min/mg protein)	1061 (43%)	
MPPGK (mg/g kidney)	11.1*^a^*	28.4 (21%)*^b^*
Scaled CL_int,u,UGT,HKM_ (ml/min/g kidney)	11.8 (43%)	30.2 (53%)
Kidney:liver ratio for scaled CL_int,u,UGT_*^c^*	1.26 (43%)	3.24 (53%)
Kidney weight (g/kg body weight)	4.5	3.1
f_u,p_	0.01	0.01
R_B_	0.6	0.6
Q_R_ (ml/min/kg)	16.4	13.2
Predicted CL_R,met,UGT_ (ml/min/kg)	0.83 (41%)	1.35 (47%)
Kidney: liver ratio for predicted CL_met,UGT_	0.29 (41%)	0.47 (47%)
Predicted CL_UGT_ (mL/min/kg)*^d^*	3.70 (9%)	4.21 (15%)
Mean predicted/observed CL_UGT_*^e^*	0.93 (9%)	1.06 (15%)

^*a*^ 11.1 mg/g kidney used for all donors, calculated as weighted (by donor number) mean of values recently reported for mixed kidney and unspecified kidney region (Al-Jahdari et al., 2006; Knights et al., 2016).

^*b*^ Donor-specific MPPGK values measured in the current study used.

^*c*^ CL_int,u,UGT,HLM_ was 9.32 ml/min/g liver, which is based on in vitro measurements in the presence of BSA (Gill et al., 2012).

^*d*^ CL_h,met,UGT_ (2.86 ml/min/kg) calculated per Gill et al. (2012).

^*e*^ Observed CL_UGT_ was 3.97 ml/min/kg (Gill et al., 2012).

Underprediction of mycophenolic acid CL_UGT_ was observed when only the hepatic contribution to glucuronidation clearance was considered (Fig. 7B). Accounting for both the hepatic and renal contributions improved the prediction of CL_UGT_ for both Scenario 1 and 2. Whereas for Scenario 1, uniformity in glucuronidation activity throughout kidney is assumed (common assumption in the literature (Gill et al., 2012; Knights et al., 2016), Scenario 2 has the assumption that glucuronidation occurs only in cortex (by applying cortex tissue weight and blood flow in the well stirred kidney model). Predicted CL_UGT_ was approximately 15% greater in Scenario 2 compared with Scenario 1 (Table 5), as demonstrated in the respective predicted/observed ratios (Scenario 1: 0.93; Scenario 2: 1.06) (Fig. 7B). Application of the cortical MPPGK obtained in the current study for the whole kidney (in conjunction with kidney weight and blood flow) would increase the predicted CL_R,met,UGT_ by 43% compared with that of Scenario 2.

## Discussion

Microsomal and cytosolic protein contents in tissues of human and preclinical species are used as scaling factors for IVIVE of microsomal metabolism data to predict drug in vivo clearance. Information on the microsomal scalar in human kidney is limited compared with the liver (Scotcher et al., 2016b). Data on the cytosolic protein in human kidney and the microsomal and cytosolic protein in preclinical species (that have explicitly accounted for protein recovery) are lacking.

In the current study, the microsomal protein content of dog kidney cortex was measured using two different microsomal protein recovery markers and compared with the corresponding values in matched liver and intestine. Further, the microsomal and cytosolic protein content was measured in 38 human kidney cortex samples. For 20 of these samples, the functional activity was assessed using a mycophenolic glucuronidation substrate depletion assay. These data were used to assess the impact of different MPPGK values, as well as different assumptions concerning the contribution of whole kidney versus only the cortex to renal drug glucuronidation, on prediction of in vivo mycophenolic acid glucuronidation clearance.

### Suitability of Microsomal and Cytosol Protein Markers for Correction of Protein Losses

Ensuring complete homogenization of kidney tissue, while also limiting contamination of microsomes with other sources of haemoproteins such as mitochondria, can be challenging. When measuring CYP content in kidney cortex, the low CYP levels and potential for spectral interference from contaminating haemoproteins make accurate quantification challenging (Jakobsson and Cintig, 1973; Ohno et al., 1982). Preliminary experiments showed minor spectral interference in the dithionite difference spectra, and therefore bias in the CYP content measurements and subsequent MPPGK estimates was unlikely when considered alongside the interassay variability (Matsubara et al., 1976). Furthermore, the dog kidney cortex microsomal CYP content measured in the current study using the dithionite difference method (Table 3) was comparable to a value reported using a customized spectral method (0.223 nmol/mg protein) (Ohno et al., 1982). CYP content measured in dog liver was also in good agreement with previously published values (Smith et al., 2008). Therefore, the standard dithionite difference spectra approach was deemed appropriate to be used in the current study.

G6Pase activity was selected as a possible alternative microsomal protein marker for correction of protein losses during centrifugation. The estimated microsomal protein recoveries in dogs using this marker (frozen tissue) were lower relative to CYP content in both liver (38% for G6Pase and 58% for CYP content) and kidney cortex (40% for G6Pase and 53% for CYP content). Subsequently, the microsomal protein content estimates were higher when using G6Pase activity. Although G6Pase is present in the nuclear envelope, it is at very low levels relative to the endoplasmic reticulum and unlikely to fully explain the marker related differences in microsomal protein content (Kartenbeck et al., 1973; Nordlie, 1979). Despite the potential for overestimation of MPPG values using G6Pase, this marker was preferred for human samples over CYP content, because of the low sensitivity of the CYP content assay and expected higher biologic variability than in dogs.

A positive correlation between G6Pase activity and mycophenolic acid CL_int,u,UGT,HKM_ was observed (Supplemental Fig. S10). Tissue storage would be an unlikely cause, as CMFT kidney cortex samples were snap-frozen within 1 h of excision. Preliminary comparisons of G6Pase activity in different batches of human kidney cortex microsomes from the same donor showed good reproducibility (Supplemental Fig. S6), confirming that the homogenization procedure was consistent. Coregulation of G6Pase and UGT enzymes is a more likely explanation for the observed correlation between G6Pase activity and mycophenolic acid CL_int,u,UGT,HKM_. Members of the hepatocyte nuclear factors families of transcription factors (HNF1 and HNF4) may be involved in regulating the expression of G6Pase (Lin et al., 1997; Rajas et al., 2002), UGT1A9 (Ramírez et al., 2008; Hu et al., 2014b), and UGT2B7 (Ramírez et al., 2008; Hu et al., 2014b). In addition, d-glucose and glucose-6-phosphate (substrate and product of G6Pase mediated reaction) and uridine-diphosphate-glucuronic acid (cofactor for UGT-mediated glucuronidation) are closely positioned in the cellular metabolic pathway (http://biochemical-pathways.com/#/map/1).

Both alcohol dehydrogenase and GST activity have been suggested as potential cytosolic protein markers (Cubitt et al., 2011). In the current study, implementation of the alcohol dehydrogenase activity assay was ineffective (data not shown). Therefore, GST activity was used as the human cytosolic protein marker, despite the presence of some GST also in the microsomes (Song et al., 2015). GST activity in human kidney cortex cytosol was higher than that in microsomes, in agreement with similar findings for human liver (Prabhu et al., 2004). Average GST activities in human kidney cortex microsomes were higher than a literature value by approximately one order of magnitude (Morgenstern et al., 1984); conversely, GST activities in human kidney cortex cytosols were on average lower than previously reported values for normal human kidney (Simic et al., 2001, 2003). Ignoring the proportion of GST activity in homogenate attributed to microsomal isoforms (14.5%) when calculating the cytosolic protein recovery would result in an increase in the average estimated CPPGK by 13%. In the extreme case, this 13% difference will contribute to potential systematic misprediction of in vivo metabolic clearance when using CPPGK as an IVIVE scaling factor for in vitro cytosolic metabolism data.

### Species and Tissue Differences in Subcellular Protein Content Estimates

The direct comparison of microsomal content of liver and kidney cortex from samples obtained from the same animals showed no correlation between the scalars, although MPPGL was on average 45% higher than MPPGK (G6Pase as marker). The mean MPPGK value in dog (44.0 mg/g kidney cortex) was higher than the corresponding value in human (26.2 mg/g kidney cortex), in agreement with literature data suggesting a similar relationship for MPPGL (Barter et al., 2007; Heikkinen et al., 2012, 2015). The variability observed in MPPGK in dogs was lower than that in humans, despite similar interassay variability in G6Pase activities, indicating greater biologic variability in human MPPGK. This trend is expected because of the higher genetic and environmental variability encountered in humans compared with that in laboratory animals.

The number of kidney cortex samples used to estimate human MPPGK in the current study (*n* = 38) was greater than the entire combined samples reported so far in the literature (*n* = 23 across four studies) (Scotcher et al., 2016b) and therefore provides a more reliable indicator of true biologic variability in this microsomal scalar. Overall, the mean MPPGK obtained here (26.2 mg/g kidney cortex) is in agreement with the value previously reported for kidney cortex microsomes (Jakobsson and Cintig, 1973), but it is more than 2-fold greater than recently reported scalars from unspecified regions or “mixed” kidney samples (Al-Jahdari et al., 2006; Knights et al., 2016). Although studies differed in microsomal protein markers used, the kidney region used is most likely the major contributor to the MPPGK differences because of higher endoplasmic reticulum content in cortex relative to medulla. This emphasizes a need for separate MPPGK scalars for cortex and whole kidney.

In addition to protein marker and kidney region, tissue source and processing were identified as important factors contributing to variability in scalars, as significant differences in MPPGK and CPPGK were found between the two sources of kidney cortex used in the current study. Demographic information, such as age, gender, and the medical history of donors was available for 20 kidney cortex samples from CMFT Biobank. This data set was insufficient for robust assessment of any potential demographic covariates of MPPGK, as reported previously for MPPGL (Barter et al., 2008). The CMFT Biobank kidney cortex samples were from donors aged 43 to 83 years at the time of nephrectomy, which represents a subsection of the overall adult population, a trend consistent with previous studies (Scotcher et al., 2016b). Further data are therefore required, particularly for younger subjects, to investigate any potential relationship between MPPGK/CPPGK and demographic factors.

The average human CPPGK (53.3 mg/kidney cortex) was approximately two-thirds of the value reported for CPPGL (Cubitt et al., 2011). To the authors’ knowledge, the potential contribution of microsomal GST isoforms within the liver homogenate was not accounted for in previous studies when GST was used as the cytosolic protein marker for liver. The estimated human S9 protein per gram of kidney cortex, based on the combined values of MPPGK and CPPGK, was 79.5 mg/g kidney cortex (24% CV), which is lower than the corresponding value for liver (121 mg/g liver), as well as an estimated value of 93.5 mg/g kidney used previously for scaling ((Nishimuta et al., 2014), calculated from an MPPGK value of 12.8 mg/g kidney and liver cytosolic recovery of 80.7 mg/g liver).

### Impact of Updated MPPGK Scaling Factors on Prediction of Renal Metabolic Clearance

As the cortex displays predominant UGT expression and greater blood flow relative to weight than medulla, it is likely that cortex has a predominant role in renal drug metabolism in vivo. For this reason, the renal cortex glucuronidation clearance of mycophenolic acid was estimated by modifying the kidney weight and renal blood flow parameters accordingly in the well stirred kidney model (Scenario 2) and compared with predictions based on assumptions of uniform kidney physiology (Scenario 1). A substantial difference was found between Scenario 1 and 2 for scaled CL_int,u,UGT,HKM_, with a less pronounced difference in the IVIVE of the overall glucuronidation clearance. In the case of mycophenolic acid, each scenario resulted in adequate prediction accuracy of its CL_UGT_ (Fig. 7B); however, scenarios differed in their estimated contribution of kidney glucuronidation relative to liver. These differences highlight the importance of knowing the source (cortex/medulla/mixed) of microsomes being used for in vitro assays and applying the correct MPPGK scalar for IVIVE of renal drug metabolism data, namely, 11.1 mg/g of kidney for mixed kidney and 26.2 mg/g of kidney for the cortex. In addition, the source of microsomes used would limit which of the available kidney models are appropriate for prediction of in vivo metabolic clearance (Fig. 1). Conversely, in vitro data required to inform parameters of a specific kidney model should be generated using microsomes prepared from the appropriate region of kidney (Fig. 1).

Mycophenolic acid is an immunosuppressant for which therapeutic drug monitoring has been proposed owing to a narrow therapeutic window and pronounced interindividual variability in its pharmacokinetics and side effects (Dong et al., 2014). Variability of approximately 50% in its CL_int,u,UGT,HKM_ observed in the current study is consistent with the interindividual variability of clearance reported clinically. Several factors have been identified as covariates of mycophenolic acid pharmacokinetics in vivo, including SNPs in UGT1A9 (e.g., −440T > C) and 2B7 (e.g., −900G > A) (Picard et al., 2005; Fukuda et al., 2012). Of the SNPs investigated in the current study, the UGT2B7 −900G > A was the only one linked with variability in mycophenolic acid in vitro CL_int,u,UGT,HKM_. This polymorphism occurs in a putative activating protein 1 binding site in the UGT2B7 promotor and could therefore affect the activity of the promotor (Hu et al., 2014a), contributing to interindividual variability in mycophenolic acid renal glucuronidation observed in vitro.

In conclusion, MPPGK in dogs was characterized for the first time, in addition to microsomal recoveries obtained for the liver and intestinal samples from the same animals. MPPGK estimated from frozen dog samples was lower than MPPGL, but it was greater than MPPGI, with no direct correlations between scaling factors. Human MPPGK in kidney cortex, measured in 38 donors (mean: 26.2 mg/g kidney cortex; range: 9.0–42.6 mg/g kidney cortex) was on average 2-fold higher than the literature MPPGK value commonly used for IVIVE scaling of renal metabolism data. Human CPPGK was measured for the first time, with mean and range of 53.3 and 30.6–123.2 mg/g kidney cortex, respectively. The current study indicates that microsomal and cytosolic scaling factors need to correspond to the tissue source (i.e., mixed kidney or cortex) used to prepare the subcellular fractions for in vitro assays. Therefore, commercial providers of human kidney microsomes and cytosols are expected to explicitly state the tissue region used. In addition to using the MPPGK for cortex, the IVIVE of in vitro data obtained in cortex microsomes needs to account for differences in cortex weight and blood flow relative to the whole kidney. Mycophenolic acid case study highlighted the implications of refined scaling factors and appreciation of regional differences on the prediction of renal metabolism and its contribution to overall clearance.

## Abbreviations

BSA: bovine serum albumin
CL_int,u,UGT,HKM_: unbound intrinsic clearance by glucuronidation in human kidney microsomes
CL_h,met,UGT_: hepatic glucuronidation clearance
CL_R,met_: renal metabolic clearance
CL_R,met,UGT_: renal glucuronidation clearance
CL_UGT_: overall glucuronidation clearance
CMFT: Central Manchester University Hospitals–NHS Foundation Trust
CPPGK: cytosolic protein per gram of kidney
CPPGL: cytosolic protein per gram liver
*f*_u_: fraction unbound
G6Pase: glucose-6-phosphatase
GST: glutathione-S-transferase
HNF: hepatocyte nuclear factors
HKM: human kidney microsome
IVIVE: in vitro-in vivo extrapolation
LC-MS/MS: liquid chromatography-mass spectrometry
MPPGI: microsomal protein per gram intestine
MPPGK: microsomal protein per gram of kidney
MPPGL: microsomal protein per gram of liver
PBS: phosphate-buffered saline
P_i_: inorganic phosphate
Q_h_/Q_R_: hepatic/renal blood flow
S9: 9000 g supernatant
UGT: uridine 5′-diphospho-glucuronosyltransferase
